# Stem Cell-Derived Exosomes in Wound Healing and Skin Regeneration: Emerging Therapeutic Strategies and Mechanisms

**DOI:** 10.3390/cells15100872

**Published:** 2026-05-10

**Authors:** Nithin Vidiyala, Pavani Sunkishala, Prashanth Reddy Parupathi, Dinesh Nyavanandi

**Affiliations:** 1 Small Molecule Drug Product Development, Cerevel Therapeutics, Cambridge, MA 02141, USA; nithinvidiyala@gmail.com; 2 Process Validation, PCI Pharma Services, Bedford, NH 03110, USA; sunkishalapavani@gmail.com; 3 Division of Pharmaceutical Sciences, Arnold & Marie Schwartz College of Pharmacy and Health Sciences, Brooklyn, NY 11201, USA

**Keywords:** stem cell-derived exosomes, chronic wound healing, skin regeneration, mesenchymal stem cells, nanomedicine, biomaterial integration

## Abstract

Chronic cutaneous wounds and traumatic skin injuries remain a major clinical challenge, characterized by dysregulated healing phases, high susceptibility to microbial infection, and suboptimal response to conventional therapies. Stem cell-derived exosomes (SC-Exos) have emerged as a paradigm-shifting, cell-free nanotherapeutic platform that harnesses the paracrine secretome of stem cells while avoiding the immunological and proliferative complications inherent to direct cell transplantation. Exosomes derived from diverse stem cell sources orchestrate multifactorial wound repair by modulating key cellular signaling cascades and transcriptomic programs that collectively regulate inflammation, angiogenesis, re-epithelialization, extracellular matrix (ECM) remodeling, and scar formation. Beyond their intrinsic regenerative capacity, SC Exos can be engineered using direct strategies (cargo loading, surface modification, biomaterial integration, and conjugation) and indirect approaches (genetic engineering, pretreatment, and preconditioning of parental cells), thereby enabling spatially controlled and temporally sustained exosome release at wound sites with enhanced bioavailability and therapeutic efficacy. In parallel, biomaterial-assisted delivery platforms, including hydrogels, scaffolds, and nanofibers, enhance exosome retention, stability, and controlled-release profiles within the wound microenvironment, thereby further potentiating tissue repair. This review provides a comprehensive overview of recent advances in SC Exos for wound healing and skin regeneration. We first summarize exosome biogenesis, molecular composition, and the distinctive characteristics of exosomes derived from different stem cell sources, along with preclinical evidence supporting their efficacy in cutaneous repair. We then critically examine exosome engineering strategies and biomaterial-integrated delivery systems that augment and fine-tune therapeutic outcomes. Finally, we discuss the current status of clinical trials of SC Exo-based therapies, key manufacturing and regulatory challenges, and future directions for translating these nanoscale, cell-free therapeutics into advanced, personalized wound management.

## 1. Introduction

Skin is the largest organ in the human body, which plays a crucial role in barrier protection, protecting against pathogens, temperature regulation, preventing dehydration, immune surveillance, and sensory perception [1,2,3,4]. It comprises multiple layers, namely, epidermis, dermis and hypodermis, that maintain the physiological homeostasis. Damage to the skin, whether from injury, trauma, burns, infections, surgery, or chronic diseases, can significantly disrupt these vital functions and initiate complex, timely and precisely coordinated wound healing process to restore tissue integrity. Impaired wound healing process further results in chronic wounds characterized by delayed healing, persistent inflammation, and increased risk of morbidity and mortality, presenting a huge burden on healthcare systems globally.

**Figure 1:**
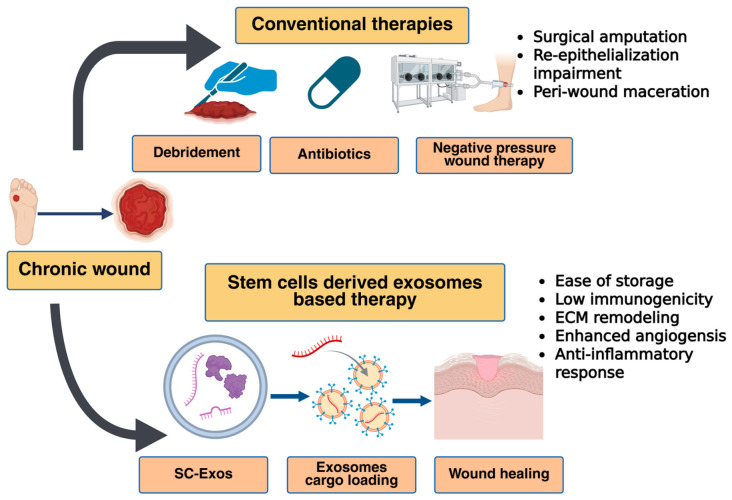
Schematic showing comparative differences between conventional wound care and stem cell-derived exosome (SC-Exos) therapy for chronic wounds. Conventional approaches, including wound debridement, advanced dressings for moisture control, and topical antimicrobials for infection management, demonstrate established clinical efficacy in reducing infection rates, promoting granulation tissue formation, and achieving wound closure in many patients. However, these therapies often provide limited capacity for modulating persistent inflammation, stimulating robust angiogenesis, or achieving scarless tissue regeneration in refractory chronic wounds, where complete healing rates remain suboptimal. In contrast, SC-Exos therapy modulates the wound microenvironment by targeting inflammation, angiogenesis, re-epithelialization, and ECM remodeling, providing an advanced regenerative solution that actively restores functional skin architecture and accelerates healing in chronic wounds. This figure was created with BioRender.com.

Chronic wounds such as diabetic foot ulcers (DFUs), pressure ulcers, and others affect approximately 2% of the global population, and this number is expected to rise due to the growing incidence of diabetes, obesity, vascular insufficiency, and increased life expectancy [1]. Traditional wound care strategies such as wound debridement, skin grafting, moist topical dressings, negative pressure wound therapy, and hyperbaric oxygen therapy provide symptomatic relief and assist in healing; however, they often fall short of addressing the root cellular and molecular defects, especially in chronic wounds (Figure 1) [3]. The limitations of conventional wound care therapies include persistent microbial biofilms and infection, hypoxia-driven impaired angiogenesis, inadequate control over chronic inflammation, insufficient extracellular matrix (ECM) remodeling, delayed or incomplete neovascularization, high recurrence rates of ulcers (especially in diabetic patients), and dependency on frequent and costly clinical interventions. Chronic wound infections promote biofilm formation and antibiotic resistance, while tissue hypoxia impairs cellular metabolism, growth factor signaling, and granulation tissue formation, all of which represent fundamental barriers that SC-Exos specifically address through antimicrobial cargo, angiogenic miRNAs, and metabolic modulation [1]. Furthermore, limited by donor site availability, infection, risk of graft rejection, and scarring in cases of large-area burns, full-thickness wounds, and ischemic ulcers, there is an urgent need to adopt advanced regenerative, targeted, and safe therapies capable of reprogramming the wound microenvironment.

In recent years, stem cell-derived exosomes (SC Exos) have emerged as a promising regenerative strategy for enhancing skin repair due to their ability to address key pathophysiological barriers in chronic wounds, including dysregulated inflammation, impaired angiogenesis, defective re-epithelialization, and aberrant ECM remodeling [1,2,5,6]. SC Exos are nanoscale extracellular vesicles secreted by various stem cell types, including mesenchymal stem cells (MSCs), induced pluripotent stem cells (iPSCs), and embryonic stem cells (ESCs), and possess regenerative and immunomodulatory functions [2,7,8,9]. They are enriched in diverse bioactive cargos, including proteins, lipids, and nucleic acids (microRNAs, long non-coding RNAs, circular RNAs, and mRNAs), as well as multiple growth factors, and have been widely investigated for wound healing and skin regeneration [1,2,8]. SC-Exos offer a potentially safer alternative to cell-based transplantation by avoiding risks such as low engraftment, uncontrolled differentiation, and tumorigenicity observed in preclinical studies, though definitive clinical safety and long-term risk profiles remain to be established through ongoing trials [10,11,12].

The role of SC Exos in wound healing and skin regeneration is multifaceted, spanning all four overlapping phases of repair: hemostasis, inflammation, proliferation, and remodeling. Mechanistically, SC Exos exert anti-inflammatory effects by promoting macrophage polarization toward the anti-inflammatory M2 phenotype, thereby attenuating chronic inflammation [2,4,13]. They deliver pro-angiogenic factors such as VEGF, angiopoietin-1, and miR-126 to endothelial cells, thereby enhancing endothelial proliferation, migration, and tube formation, and consequently accelerating neovascularization and wound closure [14]. SC Exos also stimulate keratinocyte proliferation and migration by transferring regulatory miRNAs such as miR-21, miR-31, and miR-200a, thereby promoting efficient re-epithelialization of the wound surface [15]. Furthermore, by enhancing collagen and fibronectin synthesis, modulating matrix metalloproteinases (MMPs) and their tissue inhibitors (TIMPs), and activating TGFβ/Smad signaling, SC Exos help restore the balance between ECM deposition and degradation, thereby favoring regenerative healing with reduced fibrosis [1,7].

Collectively, SC Exos orchestrate a coordinated healing response in both acute and chronic wounds through these multifactorial mechanisms, positioning them as next-generation biological therapeutics. A growing paradigm shift toward cell-free regenerative strategies has therefore placed SC Exos at the forefront of advanced wound care for skin regeneration [1,2,8,9,13]. Acting as intercellular messengers, SC Exos transfer their functional cargo to recipient cells without direct cell engraftment, thereby recapitulating many of the beneficial paracrine effects of stem cells while mitigating safety concerns. In addition, SC Exos can be engineered or enriched with specific miRNAs, proteins, or targeting ligands to enhance their specificity and functional potency for wound healing and skin regeneration [2,6,7,16]. Their incorporation into biomaterial-based delivery systems (e.g., hydrogels, microneedles, nanofibers, and scaffolds) further enables sustained, localized release and improved retention at the wound site, resulting in superior therapeutic outcomes [1,2,6,13,17]. As biological nanocarriers, SC Exos therefore represent a clinically feasible and ethically favorable alternative to cell transplantation for cutaneous repair.

Recent preclinical studies demonstrate that SC Exos accelerate wound closure, improve histological architecture, and reduce scarring in various animal models when administered as topical hydrogels or patches loaded with MSC Exos, injectable exosome formulations, or 3D bioprinted exosome-containing scaffolds [1,2,7,8,9,13,17]. Despite these encouraging data, clinical translation remains at an early stage, with only a limited number of early-phase clinical trials completed, ongoing, or planned to evaluate the safety and efficacy of SC Exos in post-surgical skin repair and chronic wound management [18]. Persistent challenges include standardization of exosome production, isolation, characterization, and quality control, as well as issues of scalability, storage stability, and regulatory compliance. Efforts to establish good manufacturing practice (GMP)-compliant workflows and to optimize storage approaches (e.g., lyophilized formulations) are addressing some of these barriers [1,2]. Additionally, integration of smart biomaterials, gene editing, pretreatment strategies, and personalized medicine concepts is anticipated to yield more targeted and effective exosome-based wound therapeutics [2].

In this review, we provide an updated overview of stem cell-derived exosomes and their recent advances in wound healing and skin regeneration as a promising cell-free therapeutic approach. We discuss exosome biogenesis, the characteristics of exosomes derived from different stem cell sources, preclinical studies across multiple wound models, engineering strategies, and diverse delivery platforms (e.g., hydrogels, scaffolds, microneedles, and nanofibers) that modulate all phases of wound healing from inflammation through remodeling. We also summarize the current clinical trial landscape and critically analyze the remaining challenges and future directions for harnessing SC Exos to achieve optimal wound repair and functional skin regeneration.

## 2. Literature Search, Study Selection, and Biogenesis of SC-Exos

### 2.1. PRISMA-Guided Literature Search and Study Selection

In accordance with the PRISMA 2020 recommendations, we conducted a structured literature search in PubMed, Scopus, and Web of Science using combinations of the terms “stem cell-derived exosomes,” “extracellular vesicles,” “chronic wound,” “skin regeneration,” and “biomaterial” without language restrictions through March 2026. Titles and abstracts were screened to exclude conference abstracts, non-original articles without primary data, studies not involving stem cell-derived exosomes, and reports unrelated to cutaneous wound healing or skin regeneration, followed by full-text assessment of the remaining records for eligibility. Priority was given to studies that provide mechanistic insight and/or quantitative outcomes, enabling comparative and analytical synthesis across stem cell sources, engineering strategies, and delivery platforms, rather than a purely descriptive summary. Given that most available evidence arises from preclinical models and a limited number of early-phase clinical trials, the clinical potential of stem cell-derived exosome-based therapies is interpreted cautiously, with explicit emphasis on heterogeneity in study design, dosing, and manufacturing, as well as current regulatory and translational barriers.

### 2.2. Biogenesis of Stem Cell-Derived Exosomes: Mechanisms and Composition

**Figure 2:**
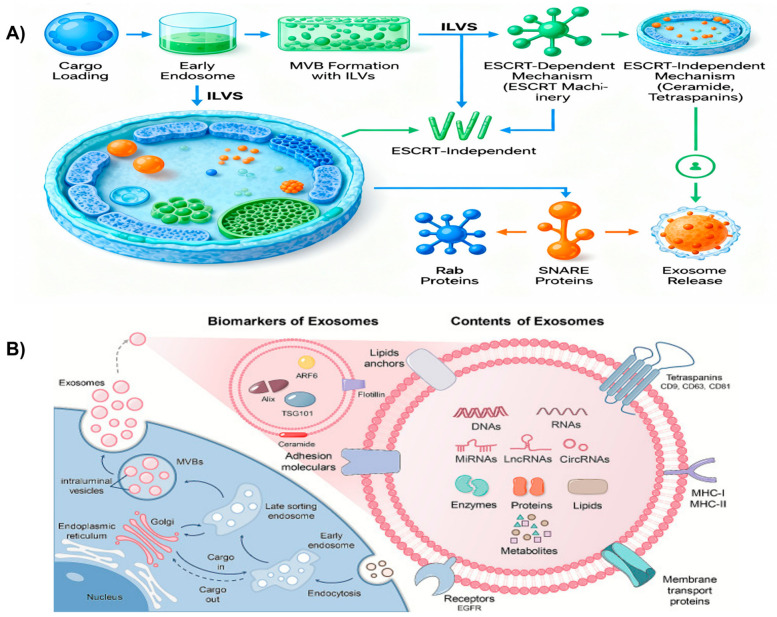
(**A**) Schematic depicting the complete exosome biogenesis pathway originating from stem cells via endosomal sorting complex required for transport (ESCRT) and ESCRT-independent mechanisms. The key cellular components include stem cell source, cargo loading, early endosomal compartments, multivesicular bodies (MVBs) populated with intraluminal vesicles (ILVs), and molecular machinery, including ESCRT complexes and Rab proteins. Created using Biorender. (**B**) Biomarkers and cargo composition of stem cell-derived exosomes. The biomarkers and composition include Alix, TSG101, ARF6 and micro RNAs (miRNAs), long non-coding RNAs (lncRNAs), circular RNAs (CircRNAs), etc. Reproduced from ref. [34] with permission from BMC. This work is licensed under a Creative Commons Attribution 4.0 (CC BY) International License.

Exosomes are nano-sized extracellular vesicles (30–150 nm) secreted by most cell types, including stem cells, that originate from the endosomal compartment. Stem cell-derived exosomes (SC Exos) have attracted particular interest as therapeutic vesicles due to their potent paracrine activity and immunomodulatory potential. They carry a cargo of bioactive molecules, such as proteins, lipids, and nucleic acids (mRNA and microRNA), and play a crucial role in intercellular communication by transferring these contents to target cells [19]. Exosomes contain conserved marker proteins such as TSG101, ALIX, HSP70/90, and tetraspanins (CD9, CD63, and CD81), along with adhesion molecules (integrins) and signaling proteins associated with angiogenesis, immune regulation, and tissue remodeling [20,21]. The lipid cargo includes sphingomyelin, phosphatidylserine, cholesterol, and ceramide, which confer membrane rigidity and stability and participate in vesicle budding [22]. Stem cell-derived exosomes are enriched in messenger RNAs, microRNAs (miRNAs), long non-coding RNAs, and circular RNAs that regulate angiogenesis, resistance to apoptosis, and immune modulation [23,24]. Understanding their biogenesis is therefore critical, as it determines which cargo is packaged, how much is released, and how these vesicles are targeted to specific recipient cells. Exosome biogenesis is broadly classified into two types: endosomal sorting complex required for transport (ESCRT)-dependent mechanisms and ESCRT-independent mechanisms such as RAB31-mediated pathways, tetraspanin-enriched microdomains, and ceramide-dependent budding (Figure 2) [19,25,26].

#### 2.2.1. ESCRT-Dependent Pathway

Exosome formation begins at the plasma membrane (PM), where membrane deformation leads to inward budding or invagination of the endosomal membrane to generate intraluminal vesicles (ILVs) within early endosomes [26,27]. Primary endosomes (ILVs) subsequently load cellular cargo via the trans-Golgi network and mature into late endosomes, which enlarge to form multivesicular bodies (MVBs) that ultimately release ILVs into the extracellular space as exosomes [28]. This process is mediated by the ESCRT machinery, in which ESCRT I and ESCRT II drive membrane deformation, while ESCRT III orchestrates vesicle scission. During inward budding of late endosomes, exosomes selectively package specific proteins and lipids into ILVs through coordinated ESCRT-dependent and ESCRT-independent sorting mechanisms. Cytoplasmic tails of transmembrane proteins are often monoubiquitinated and trafficked from the trans-Golgi network, serving as key molecular signals that direct their recognition and incorporation into ILVs during MVB biogenesis [29,30]. MVBs then undergo intracellular trafficking via either secretory or lysosomal pathways: in the secretory route, fusion of MVBs with the plasma membrane releases ILVs as exosomes into the extracellular milieu, whereas lysosomal trafficking leads to fusion with lysosomes and enzymatic degradation of ILVs by lysosomal hydrolases within the lumen. The relative formation and fate of MVBs and ILVs, governed by ESCRT components, ultimately determine the quantity and composition of exosomes released.

#### 2.2.2. ESCRT-Independent Pathway

Exosomes also form ILVs within MVBs via multiple cargo-sorting routes, independent of the canonical ESCRT machinery. Accumulating evidence supports the coexistence of ESCRT-dependent and ESCRT-independent mechanisms, including RAB31-mediated pathways, the syndecan–syntenin–ALIX axis, ceramide-dependent budding, and tetraspanin-enriched microdomains, which together enable selective cargo incorporation into ILVs even in the absence of core ESCRT subunits and thereby generate functionally diverse exosome populations. In the RAB31-mediated mechanism, RAB31 promotes exosome biogenesis by facilitating epidermal growth factor receptor (EGFR) phosphorylation and activation within MVBs to drive ILV formation [31]. Here, lipid raft microdomains associate with flotillin proteins to support the biogenesis process. In addition, RAB31-mediated recruitment of TBC1D2B inhibits RAB7 activity, preventing MVB–lysosome fusion and thereby avoiding ILV degradation [29]. In the syndecan–syntenin–ALIX axis, syndecan 1 and syntenin 1 recruit the ALIX–ESCRT III complex to enable ubiquitination-independent sorting of specific cargoes into ILVs [32,33].

Another ESCRT-independent route is ceramide-dependent budding, in which sphingomyelinase activity and ceramide-induced negative membrane curvature promote ILV formation [34]. Furthermore, tetraspanin-enriched microdomains cluster specific cargoes such as CD9, CD63, and CD81 in a ubiquitination-independent manner [26]. Overall, the biogenesis of stem cell-derived exosomes is a tightly regulated, multistep process involving both ESCRT-dependent and ESCRT-independent pathways. A deeper understanding of these mechanisms of exosome formation and release not only elucidates fundamental aspects of stem cell biology but also opens new avenues for exploiting SC Exos as therapeutic agents in regenerative medicine and beyond.

Finally, the yield and molecular composition of stem cell-derived exosomes are strongly influenced by both upstream culture conditions and downstream isolation strategies. Stem cell-specific factors such as hypoxia, three-dimensional cultures, and developmental stage, as well as the choice of isolation method, can markedly affect exosome yield, purity, and bioactivity [35,36,37,38,39,40,41,42,43]. Isolation methods must therefore be selected carefully to balance yield and purity while preserving vesicle integrity and functional cargo for the intended application. Widely used approaches include differential centrifugation, size-exclusion chromatography (SEC), precipitation, ultrafiltration, immunoaffinity capture, and emerging microfluidic/lab-on-a-chip techniques, each with distinct advantages and limitations [44,45]. A comprehensive comparison of these methods, based on key performance metrics relevant to wound healing applications, including yield, purity, scalability, cost, and impact on therapeutic function, is presented in Table 1. The selected method influences not only yield and cargo integrity but also downstream data interpretation due to trade-offs between purity and throughput, variability in measurement modalities, and differences in stem cell sources and culture conditions (e.g., hypoxia and inflammatory priming), making method selection critical for translational applications [41,46]. At present, no single isolation technique can be considered universally “best,” as each balances yield, purity, scalability, and cost in different ways. Consequently, many researchers employ combination or sequential methods and seek to standardize upstream culture and isolation workflows to accelerate clinical translation of SC Exos, making data more interpretable and studies more reproducible [41]. Combination workflows such as precipitation followed by SEC or ultrafiltration with tangential flow filtration often provide the best compromise for clinical-grade SC Exos by improving reproducibility and reducing contaminants while maintaining bioactivity [20,44,45]. Upstream culture conditions (e.g., hypoxia, 3D culture, and inflammatory priming) also modulate exosome yield and cargo and should be optimized in tandem with the isolation strategy to maximize therapeutic performance in wound healing [44,45]. These considerations underscore the need for standardized, GMP-compliant protocols to enable consistent clinical outcomes.

## 3. Stem Cells as Sources of Therapeutic Exosomes

Stem cell-derived exosomes, particularly those from mesenchymal stem cells (MSCs), have emerged as one of the most promising therapeutic exosome sources due to their capacity to modulate cell signaling, regulate immune responses, and enhance tissue repair and angiogenesis, making them effective in treating conditions ranging from chronic wounds to various inflammatory skin diseases [23,47,48,49,50,51]. MSCs obtained from different tissues—such as bone marrow, adipose tissue, placenta, umbilical cord tissue, and dental pulp—are enriched with regenerative bioactive molecules, including long non-coding RNAs (lncRNAs), which foster a regenerative rather than fibrotic environment during wound healing and skin regeneration [52,53]. In addition to MSCs, other stem cell sources such as induced pluripotent stem cells (iPSCs) and epidermal stem cells (EpSCs) also deliver unique molecular cargos and have demonstrated significant potential for wound healing and skin regeneration. This section discusses various stem cell sources and summarizes preclinical studies that promote wound healing through diverse mechanisms and by activating distinct cell signaling pathways.

### 3.1. Mesenchymal Stem Cells (MSCs)-Derived Therapeutic Exosomes

**Figure 3:**
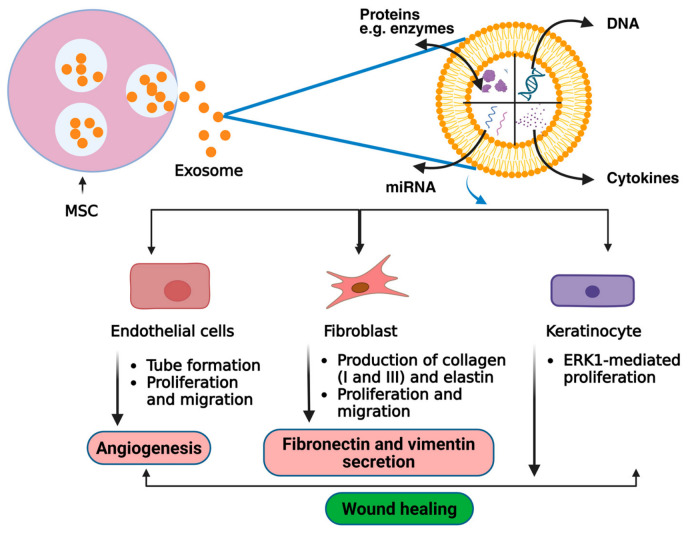
Different mechanisms of wound healing by MSCs-derived exosomes. MSCs-Exos cargo is rich in different RNAs, miRNAs, proteins, and cytokines that promote wound healing through multiple mechanisms, viz., enhancing keratinocyte proliferation, collagen and elastin remodeling, and neovascularization. This figure was created with BioRender.com.

MSCs-derived exosomes (MSC-Exos) are versatile nanovesicles that recapitulate many of the therapeutic properties of their parental cells and play a pivotal role in multiple stages of chronic wound healing. [11]. Depending on their tissue of origin, MSC-Exos are broadly classified into bone marrow MSCs (BMMSCs), adipose-derived MSCs (ADMSCs), umbilical cord MSCs (UCMSCs), placental MSCs, and dental pulp MSCs. These exosomes facilitate intercellular communication by delivering functional lncRNAs, DNA, proteins, and metabolites to wound sites, thereby promoting tissue repair through anti-inflammatory effects, improved perfusion, and intrinsic targeting capacity [7,52,54,55,56,57]. MSC-Exos stimulate fibroblast, keratinocyte, and human umbilical vein endothelial cell proliferation and migration, enhance the production of wound healing proteins, including fibronectin and vimentin, promote angiogenesis, and regulate extracellular matrix (ECM) remodeling. Among the various MSC sources, BMMSCs-Exos, ADMSCs-Exos, and UCMSCs-Exos account for a substantial proportion of the exosomes investigated in preclinical studies for wound healing and skin regeneration. Importantly, the therapeutic profile of MSC-Exos varies by source, reflecting differences in proteomic and RNA cargo composition. For example, BMMSCs-Exos, ADMSCs-Exos, and UCMSCs-Exos contain distinct protein repertoires that support integrin binding, cell adhesion, and immune regulation, respectively (Figure 3).

Among these, BMMSCs-Exos, ADMSCs-Exos, and UCMSCs-Exos constitute a significant proportion of MSC-Exos in wound healing and skin regeneration, as revealed by various preclinical studies [7,10,52,58,59,60]. Common surface markers expressed by MSC-Exos include CD29, CD44, CD73, TSG101, CD81, CD63, CD9, HSP60, HSP70, HSP90, and ALIX. In addition, these vesicles are enriched in growth factors such as PDGF-BB, VEGF-A, hepatocyte growth factor, and FGF-2, which contribute to wound repair by supporting angiogenesis, fibroblast activation, and matrix synthesis. Comparative studies have shown that ADMSCs-Exos and UCMSCs-Exos more strongly stimulate keratinocyte proliferation, whereas BMMSCs-Exos tend to exert a greater effect on dermal fibroblast proliferation at equivalent doses. In another study, BMMSCs-, ADMSCs-, and UCMSCs-derived exosomes all accelerated wound closure, with UCMSCs-Exos showing particularly strong effects on angiogenesis and collagen deposition [61,62,63]. A side-by-side comparative analysis of the mechanistic details of BMMSCs Exos, ADMSCs Exos, and HucMSCs Exos for cutaneous wound repair is presented in Table 2.

#### 3.1.1. Bone Marrow MSCs (BMMSCs)-Derived Therapeutic Exosomes

Bone marrow-derived mesenchymal stem cells (BMMSCs) are among the most extensively investigated stem cell types in clinical research because of their relative accessibility, low immunogenicity, and broad involvement across multiple phases of wound repair [61,64,66]. BMMSCs-derived exosomes (BMMSCs Exos) function as key paracrine mediators that accelerate wound healing, including diabetic cutaneous wounds, mainly through robust pro-angiogenic and pro-regenerative effects, as further summarized in Table 2. Mechanistically, BMMSCs Exos deliver long non-coding RNA (lncRNA) cargos such as KLF3 AS1, which upregulates VEGFA expression via specific miRNA interactions and thereby enhances angiogenesis and diabetic wound repair [71]. Their protein cargo is enriched in multiple growth factors and signaling molecules—HGF, IL-6, IGF1, SDF1, NOTCH2, ADAM10, and CACNA2D1—that collectively activate critical pathways in target cells to support proliferation, migration, and tissue remodeling [72,73]. In HaCaT keratinocytes and human dermal fibroblasts (HDFs), BMMSCs-Exos promote full-thickness cutaneous wound healing by suppressing TGF-β/Smad signaling. They downregulate pro-fibrotic mediators (TGF-β1, Smad2, Smad3, and Smad4) and upregulate anti-fibrotic TGF-β3 and Smad7, thereby rebalancing ECM remodeling and inflammation resolution [66]. Beyond proteins, BMMSCs Exos carry diverse miRNAs, lncRNAs, and circular RNAs (circRNAs) that converge on multiple wound-relevant pathways [74]. For example, exosomal miR 93 3p promotes proliferation and migration of H_2_O_2_-injured HaCaT cells by downregulating the APAF1 axis [75], whereas miR-223 reduces accumulation of pknox1 and accelerates healing by driving macrophage polarization from pro-inflammatory M1 to pro-regenerative M2 phenotype in murine models [76].

Overexpression of miR 126 in BMSCs elevates VEGF and Ang 1 levels and activates PIK3R2/PI3K/AKT signaling in HUVECs, thereby augmenting angiogenesis [14]. Likewise, upregulated miR-21-5p and miR-1260a in magnetically pretreated BMMSCs Exos activate PI3K/AKT and ERK1/2 pathways, leading to enhanced angiogenesis, reduced scar width, and faster wound closure [77,78]. Long non-coding RNAs such as H19 and KLF3 AS1 in BMMSCs Exos further stimulate fibroblast and HUVEC proliferation and migration via VEGFA upregulation and PI3K/AKT pathway activation, particularly in diabetic wound models [71,79]. In addition, BMMSCs Exos circRNAs can activate Nrf2 signaling by recruiting TAF15, thereby promoting angiogenesis and accelerating healing in diabetic foot ulcers [64]. Preconditioning strategies further amplify the therapeutic potency of BMMSCs-Exos. Melatonin-pretreated BMMSCs yield exosomes that enhance HUVEC proliferation and migration, upregulate miR-221-3p, and activate the AKT/eNOS pathway, thereby improving tube formation and wound repair in diabetic mice compared with untreated BMMSCs-Exos [80,81]. Similarly, empagliflozin-pretreated BMMSCs generate exosomes (PGZ Exos) with superior angiogenic capacity; these exosomes activate PI3K/AKT/eNOS signaling, increase endothelial proliferation and tube formation, upregulate pro-angiogenic factors, including VEGF, and ultimately accelerate diabetic wound closure with improved ECM remodeling [82]. Despite these encouraging mechanistic and preclinical data, BMMSCs Exos still require further investigation to fully elucidate their effects in chronic wounds and to enable robust clinical translation.

#### 3.1.2. Adipose MSCs (ADMSCs)-Derived Therapeutic Exosomes

**Figure 4:**
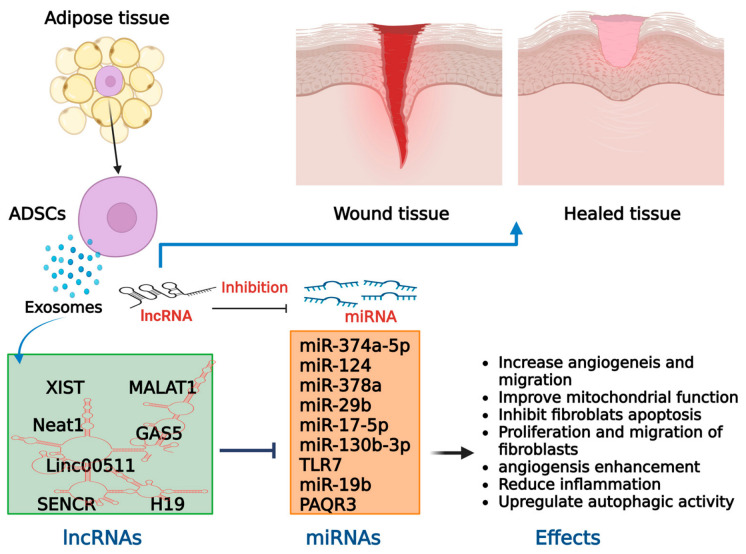
Wound healing by exosomes derived from adipose-derived mesenchymal stem cells (ADMSCs) carrying long non-coding RNAs (lncRNAs) through delivering regulatory lncRNAs to wound tissue. The figure shows exosomes secreted by ADMSCs containing lncRNAs (Neat1, XIST, MALAT1, GAS5, H19, SENCR, and Linc00511) that are delivered to wound tissue, resulting in accelerated regeneration and transition from wounded to healed skin. The lower panel details the molecular mechanisms by which individual lncRNAs and their associated miRNAs and target pathways modulate key wound healing processes—such as inhibition of fibroblast apoptosis, autophagy, migration, proliferation, regulation of inflammatory gene expression, and extracellular matrix remodeling promotion of angiogenesis for efficient wound healing and tissue repair. Reproduced from ref [23] with permission from Springer Nature. This work is licensed under a Creative Commons Attribution 4.0 (CC BY) International License.

ADMSCs-derived exosomes (ADMSCs Exos) are widely used in wound healing and skin repair because adipose tissue is easily accessible, yields large cell numbers, and exhibits low immunogenicity and favorable safety profiles. These exosomes are enriched in proteins, miRNAs, lncRNAs, and circRNAs that together modulate multiple phases of the wound healing cascade—including cell proliferation and migration, angiogenesis, inflammatory resolution, and ECM remodeling—thereby enhancing overall healing efficacy (Figure 4) [83,84,85,86]. Mechanistically, ADMSCs Exos activate WNT/β catenin signaling to drive fibroblast proliferation and migration, stimulate endothelial cells to support neovascularization, and regulate collagen synthesis and fibroblast differentiation to limit scar formation, while balancing pro- and anti-inflammatory cytokines to create a pro-healing microenvironment [85,86,87,88]. Multiple studies confirm that ADMSCs Exos promote skin regeneration and wound closure through coordinated regulation of these cellular processes [2,8,42,87,88,89,90]. Furthermore, to augment tissue repair and wound healing, the intrinsic targeting properties of ADMSCs can be engineered employing diverse strategies.

At the cellular level, ADMSCs’ exos increase HDF proliferation and migration, attenuate inflammatory responses, and support ECM remodeling, resulting in accelerated wound closure and improved re-epithelialization [91,92]. In both healthy and diabetic mouse models, they promote M2 macrophage polarization and downregulate pro-inflammatory cytokines such as TNF-α and IL-6, which enhances proliferation, collagen deposition, and neovascularization [13]. Biomaterial-assisted delivery further improves their performance: ADMSCs Exos loaded into hydrogels, nanofiber scaffolds, collagen matrices, or human acellular amniotic membrane (hAAM) scaffolds provide sustained local release, reduce inflammation, increase M2 macrophage recruitment, and support superior new skin formation in diabetic wounds [93]. Collectively, these strategies highlight the value of ADMSCs Exos as versatile, engineerable platforms for skin repair [94].

Non-coding RNA cargos are central to the functional versatility of ADMSCs Exos. Exosomal miRNAs such as miR-34a-5p, miR-21-5p, miR-124-3p, miR-125a-3p, and miR-146a-5p enhance proliferation and migration of fibroblasts, HaCaT keratinocytes, and HUVECs, while driving M2 macrophage polarization and angiogenesis via PTEN inhibition and activation of pro-healing pathways [89,95,96,97]. Hypoxic preconditioning of ADMSCs reshapes exosomal miRNA profiles—decreasing miR-99b and miR-146a while increasing miR-21-3p, miR-126-5p, and miR-31-5p—to further potentiate wound healing [87]. Additional miRNAs such as miR-486-5p, miR-125a, and miR-31 promote neovascularization and collagen production through PI3K/AKT signaling [50,98], whereas miR 194-enriched ADMSCs Exos directly target the TGF-β1 promoter to reduce IL-1β and IL-6, suppress TGF-β1 induced fibrosis, and mitigate excessive scarring [50,89], whereas miR-194 enriched ADMSCs Exos directly target the TGF-β1 promoter to reduce IL-1β and IL-6, suppress TGF-β1-induced fibrosis, and mitigate excessive scarring [58]. ADMSCs Exos also deliver lncRNAs and circRNAs that fine-tune inflammatory and reparative signaling. Delivery of lncRNA H19 via ADMSCs Exos regulates miR-130 b 3p, promoting M2 macrophage polarization, fibroblast proliferation and migration, and angiogenesis [99]. In human skin fibroblasts, H19-containing ADMSCs Exos enhance proliferation, migration, and invasion by activating Wnt/β catenin signaling and downregulating miR-19 b and SOX9, thereby accelerating cutaneous wound healing in mice [98]. Exosomal early growth response 1 (EGR 1) can bind to the lncRNA SENCR promoter, increase VEGF A expression, and promote wound repair through the EGR 1/lncRNA SENCR/DKC1/VEGF A axis [65]. CircRNA engineering further expands these effects: mmu_circ_0001052-modified ADMSCs Exos downregulate miR-106a-5p and activate the FGF4/p38MAPK pathway, enhancing angiogenesis and reducing apoptosis in diabetic foot ulcer models [100]; circRps5-overexpressing ADMSCs Exos decrease neutrophil infiltration and CRP levels under hypoxia, alleviate chronic inflammation, and improve wound healing in diabetic models [90]. Overall, ADMSCs Exos provide a potent, cell-free platform that simultaneously supports fibroblast and keratinocyte activity, angiogenesis, collagen remodeling, and dermal matrix reconstruction by delivering coordinated miRNA, lncRNA, and circRNA cargos and engaging key pathways such as PI3K/AKT, Wnt/β catenin, and ERK/MAPK.

Human umbilical cord mesenchymal stem cell-derived exosomes (HucMSCs Exos) represent a particularly promising cell-free therapeutic option for accelerating wound healing and promoting skin regeneration across all phases of repair [101]. Their advantages include high proliferative capacity for large-scale production, low immunogenicity, non-invasive sourcing from discarded perinatal tissue, and rich growth factor content, making them attractive for treating chronic wounds, UV-damaged skin, burns, and surgical injuries. HucMSCs Exos attenuate excessive inflammation, enhance fibroblast and keratinocyte proliferation and migration in a dose-dependent manner, and facilitate ECM remodeling, collectively increasing wound closure rates and improving tissue quality [101]. They also deliver angiopoietin 2, FGF-2, VEGFA, and PDGF-BB to endothelial cells, thereby driving robust neovascularization [68]. Key signaling pathways activated by HucMSCs Exos in wound repair include Wnt/β catenin, PI3K/AKT, and ERK1/2, which coordinate balanced collagen deposition, re-epithelialization, and granulation tissue formation [59].

In preclinical models, topical or subcutaneous administration of HucMSCs Exos consistently results in faster wound closure, superior collagen organization, enhanced neovascularization, and reduced scar formation [102,103]. HucMSCs Exos not only recruit fibroblasts but also promote skin nerve fiber regeneration by increasing secretion of nerve growth factors, contributing to improved functional recovery alongside structural repair [102]. Additional studies report elevated chemokine expression, increased M2 macrophage polarization, VEGF upregulation, and TNF α downregulation, all of which accelerate wound healing and improve tissue quality [68,103].

As with BMMSCs and ADMSCs Exos, non-coding RNAs are central to HucMSCs Exos function. HucMSCs Exos highly express miR 21 5p, miR 150 5p, and miR 125b 5p, which attenuate apoptosis in HUVECs and inhibit TP53INP1 and TGFBR1/2, promoting anti-myofibroblast differentiation and activating PI3K/AKT signaling via PTEN suppression to improve wound healing outcomes [14,104]. CircHIPK3-containing HucMSCs Exos enhance vascularization in diabetic wounds by targeting and downregulating miR 20b 5p, leading to increased Nrf2 and VEGFA expression [105]. These exosomes are also enriched in plasminogen activator inhibitor 1, which modulates plasminogen activation and fibrosis to support more favorable healing [59]. Genetic engineering approaches have produced HucMSCs Exos enriched with eNOS (HucMSCs Exos/eNOS) that protect HUVECs from apoptosis and enhance their function by activating PI3K/AKT/mTOR and FAK/ERK1/2 pathways; higher eNOS levels correlate with reduced apoptosis, increased microvessel formation, and faster wound closure [106]. HucMSCs Exos display particularly strong efficacy in diabetic wound models, where they suppress macrophage pyroptosis, reduce oxidative stress, and accelerate wound closure. Multiple studies show that HucMSCs Exos attenuate oxidative injury in HaCaT keratinocytes and HUVECs in vitro and improve diabetic wound healing in vivo by inhibiting H_2_O_2_-induced apoptosis through suppression of apoptosis-inducing factor (AIF) nuclear translocation [106,107,108,109]. Enhanced vascular remodeling and epidermal re-epithelialization are consistently observed in these models. Although most evidence currently derives from animal studies, the compelling preclinical data underscore the need for well-designed human clinical trials to clarify the therapeutic benefits and safety profile of HucMSCs Exos in skin wound repair and regeneration.

#### 3.1.3. Placental MSCs (PMSCs)-Derived Therapeutic Exosomes

Placental mesenchymal stem cells (PMSCs) are MSCs derived from different regions of the highly vascularized placenta [110]. Although less accessible than ADMSCs or BMMSCs, PMSCs demonstrate superior proliferative capacity, ethical acceptability, and long-term expansion potential [48,111]. PMSC-derived exosomes (PMSCs-Exos), enriched with diverse bioactive molecules, represent promising tools for regenerative medicine, particularly wound healing and skin tissue repair.

PMSCs-Exos enhance regenerated skin quality, reduce fibrosis, accelerate wound closure, and promote restoration of skin appendages (hair follicles and sebaceous glands) through inflammation modulation, balanced collagen synthesis/remodeling, and downregulation of fibrotic pathways [112]. Topical or local PMSCs-exos administration improves skin texture and elasticity with minimal risk of immune rejection, establishing them as a compelling cell-free therapeutic strategy for both acute and chronic skin wounds [112]. In a rat chronic wound model, peri-wound injection of PMSCs-Exos significantly accelerated wound closure and promoted skin appendage regeneration. These effects were mediated by downregulation of Yes-associated protein (YAP) expression and inhibition of EN1 activation, key regulators of fibroblast activation and scar formation, thereby facilitating tissue regeneration, mitigating fibrosis, and improving functional wound healing outcomes [112].

#### 3.1.4. Dental Pulp MSCs-Derived Therapeutic Exosomes

Dental pulp stem cell (DPSC)-derived exosomes, rich in bioactive cargo, offer a promising cell-free therapeutic approach for skin tissue regeneration and accelerated cutaneous wound healing across multiple repair phases [113]. Their primary mechanism involves activation of the Cdc42/p38 MAPK signaling pathway, which significantly enhances angiogenesis and accelerates wound closure in preclinical full-thickness wound models. Key advantages of DPSC-Exos include low immunogenicity, accessible dental pulp tissue, ease of storage/transport, and ability to cross biological barriers for targeted delivery [114].

Zhou et al. demonstrated that DPSC-Exos treatment significantly increased CD31-positive blood vessel expression and neovascularization at wound sites, accompanied by enhanced HUVEC proliferation, migration, and capillary tube formation [115]. Beyond angiogenesis, DPSC-Exos promoted collagen deposition, re-epithelialization, and elevated PCNA-positive cell expression, indicating coordinated inflammation resolution and cell proliferation. In diabetic wound models, DPSC-Exos ameliorated oxidative stress, enhanced angiogenesis, and accelerated healing [114]. DPSC-Exos applications extend beyond cutaneous wounds to include dental pulp regeneration and periodontal repair via M1-to-M2 macrophage polarization, modulation of IL-6/JAK2/STAT3 signaling, and improved flap survival in reconstructive surgery [116,117]. Overall, DPSC-Exos represent a transformative cell-free platform for chronic wounds, diabetic ulcers, and tissue regeneration through the promotion of angiogenesis, modulation of inflammation, enhanced proliferation/migration, and organized tissue remodeling.

### 3.2. Studies on the Cellular and Molecular Mechanisms of SC-Exos

SC-Exos orchestrate wound repair through targeted modulation of key signaling pathways, validated across cell-based (in vitro) and preclinical (in vivo) experimental systems (Table 3). A representative list of SC-Exos from different sources for wound healing and skin regeneration in both in vitro and in vivo models is presented in Table 3. These mechanisms span all wound healing phases, viz., inflammation, proliferation, and remodeling, and underscore the therapeutic rationale for clinical translation. The major mechanisms include anti-inflammatory effects, angiogenesis promotion and re-epithelialization and ECM remodeling. In the context of anti-inflammatory effects, MSC-Exos promote M2 macrophage polarization in RAW264.7 cultures by delivering miR-146a/let-7b, which suppresses TNF-α/IL-6 while upregulating IL-10/Arg-1 [76]. Similarly, in diabetic mouse wounds, UCMSC-Exos shorten inflammation via exosomal IL-10 mRNA and miR-125b transfer, accelerating proliferative healing [105]. Angiogenesis promotion is also facilitated by SC-Exos for wound healing applications.

BMMSC-Exos activate VEGF/miR-126 signaling via PI3K/AKT and ERK1/2 pathways in HUVECs, enhancing endothelial proliferation, migration, and tube formation [64]. DPSC-Exos stimulate Cdc42/p38 MAPK in db/db mouse wounds, increasing CD31^+^ vessels [113]. ADMSC-Exos upregulate miR-21-3p/miR-31-5p to activate Wnt/β-catenin signaling in vitro and in porcine models [109]. Furthermore, re-epithelialization and ECM remodeling also play pivotal roles in accelerated wound healing using SC-Exos. UCMSC- and iPSC-Exos deliver miR-21/miR-31/miR-200a to HaCaT keratinocytes, activating ERK1/2 and Wnt/β-catenin pathways for re-epithelialization [103,107,118]. BMMSC-Exos downregulate TGF-β1/Smad2/3 and upregulate TGF-β3/Smad7 in HDFs, balancing collagen deposition and reducing scarring [66]. PMSC-Exos inhibit YAP/EN1 signaling in rat wounds, limiting fibrosis while promoting appendage regeneration [112]. These conserved mechanisms across SC-Exos sources highlight their therapeutic potential, pending clinical validation in human wounds.

### 3.3. Induced Pluripotent Stem Cells (iPSCs)-Derived Therapeutic Exosomes

Among emerging stem cell-derived exosomes, iPSCs-derived exosomes (iPSCs Exos) offer a potent cell-free therapeutic strategy for enhancing wound closure and skin regeneration by delivering regenerative cargo to resident skin cells [119]. iPSCs Exos are rich in pro-angiogenic microRNAs (e.g., miR-21-5p) and growth factors that promote wound healing and skin regeneration by downregulating PTEN and activating the PI3K/AKT and ERK1/2 signaling pathways [120]. Activation of these pathways in dermal fibroblasts and endothelial cells enhances keratinocyte migration, fibroblast proliferation, capillary tube formation, and neovascularization [121]. In an earlier study, exosomes derived from autologous monkey iPSCs significantly accelerated wound healing [122]. Another preclinical study reported marked resolution of inflammation and reduced wound area in diabetic mice treated with iPSC-derived exosomes, highlighting iPSC-derived exosomes as reproducible and scalable biomanufacturing products [123]. Beyond potentiating angiogenesis, iPSC-derived exosomes facilitate keratinocyte proliferation and migration, resulting in approximately a 30% increase in keratinocyte coverage of wound beds after 7 days of healing through modulation of ERK1/2 and Wnt/β-catenin signaling [124]. Jiang et al. demonstrated that iPSCs Exos attenuated inflammation and shortened the inflammatory phase by promoting macrophage polarization toward the pro-regenerative M2 phenotype via exosomal delivery of IL-10 mRNA and miR-125 b [55]. In a recent report, topical or injectable administration of human iPSCs-derived exosomes significantly accelerated re-epithelialization and granulation tissue formation, leading to approximately 25% faster wound closure and improved collagen I:III ratios in full-thickness diabetic wound models; these changes were associated with enhanced collagen remodeling and reduced scar formation [121]. Furthermore, iPSCs Exos enriched in antioxidant enzymes and Nrf2 activating microRNAs ameliorated the impaired healing environment characteristic of diabetes by reducing oxidative stress, restoring redox balance, promoting neovascularization, and suppressing excessive inflammation through PTEN downregulation and increased Nrf2 activity in diabetic wounds [121,124]. Overall, iPSCs Exos represent a scalable, immunologically safe modality for both acute and chronic wounds and regenerative dermatology, owing to their robust pro-angiogenic, antioxidant, anti-inflammatory, and ECM-modulating cargos, making them a promising next-generation cell-free therapy for wound repair.

### 3.4. Epidermal Stem Cells (EpSCs)-Derived Therapeutic Exosomes

Epidermal stem cells (EpSCs)-derived exosomes (EpSCs Exos) have recently attracted attention as a specialized cell-free therapeutic modality for cutaneous wound healing and scar modulation. Localized in the basal layer of the epidermis and hair follicle bulge, EpSCs are critical for epidermal homeostasis, barrier maintenance, and regeneration following injury, and their exosomes recapitulate many of these functions through paracrine signaling. EpSCs Exos deliver a repertoire of bioactive cargos—including proteins, cytokines, and non-coding RNAs—that collectively regulate keratinocyte and fibroblast activity, modulate inflammation, and remodel the extracellular matrix to favor regenerative healing [125,126,127]. Preclinical studies have demonstrated that EpSCs Exos promote endothelial cell survival in diabetic wound microenvironments by alleviating excessive autophagy-induced apoptosis and protecting vascular endothelial cells from oxidative stress, thereby improving angiogenesis and accelerating wound closure [125]. In human skin fibroblasts (HSFBs), EpSCs Exos enhance collagen synthesis and favor a more regenerative collagen III/I ratio through phosphorylation of PKN1 and regulation of cyclin expression, leading to dose-dependent improvements in wound repair [128]. In full-thickness wound models, EpSCs Exos downregulate TGF β1 and associated pro-fibrotic signaling, resulting in accelerated wound healing, reduced scar formation, enhanced angiogenesis, and improved ECM composition; these effects are accompanied by better regeneration of cutaneous appendages such as hair follicles and sebaceous glands [129]. In another preclinical model, modulation of the dermal microenvironment to favor repair over fibrosis was demonstrated through the topical application of EpSCs-Exos through the enhancement of keratinocyte proliferation and migration and accelerating re-epithelialization by activating PI3K/Akt and Wnt/β-catenin pathways and JAK2/STAT3 signaling pathway [125,128]. For clinical translation, EpSCs-Exos can be incorporated into biocompatible delivery systems, such as hydrogels, collagen scaffolds, or nanofiber dressings, to improve wound healing kinetics by providing sustained release and enhanced retention at the wound site. In an earlier preclinical study, EpSCs-Exos-loaded gelatin methacryloyl hydrogel showed reduced dressing change frequency and improved wound healing [130]. Together, these findings suggest that EpSCs Exos represent a promising, epidermis-focused exosome source for promoting high-quality skin regeneration with minimized fibrosis.

## 4. Engineering Strategies for Boosting the Efficacy of Exosomes

**Figure 5:**
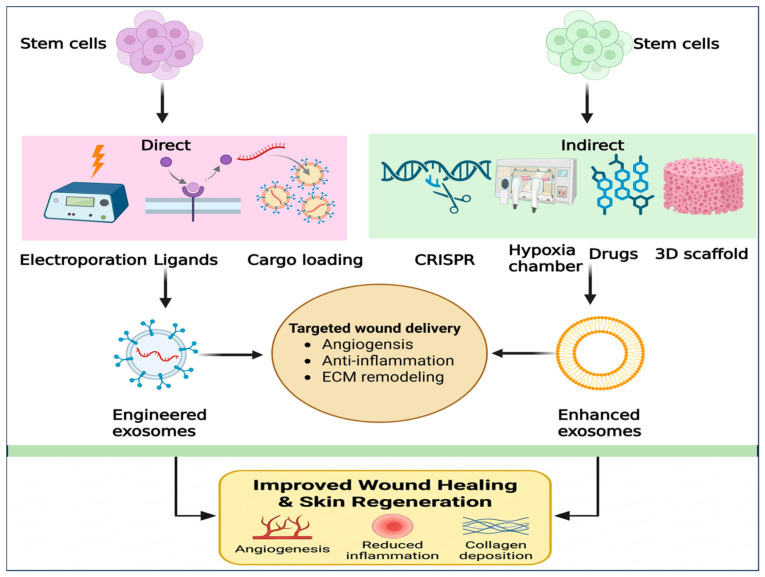
Different strategies for engineering exosomes through direct and indirect approaches for improving wound healing and skin regeneration. This figure was created with BioRender.com.

Natural SC Exos promote wound healing by modulating key biological processes, including cell proliferation, migration, inflammation, and angiogenesis. However, their clinical applications still face challenges, including batch-to-batch variability, differences in extraction methods, and inter-individual donor variability, all of which can limit therapeutic effectiveness and consistency in wound therapy [2,85]. To address these limitations, substantial efforts have focused on engineering exosomes to augment and refine their functions, broadly through two complementary approaches: direct exosomal modification and indirect modification via parental cell preconditioning and pretreatment (Figure 5) [131,132,133]. Direct exosome engineering strategies encompass the fabrication or modification of exosomes using techniques such as electroporation, sonication, freeze–thaw cycling, and other surface modifications, as well as advanced cargo loading and targeting approaches, often combined with functional biomaterials [2,131]. Indirect strategies include genetic or environmental manipulation of parental cells (e.g., gene editing, hypoxia, and pharmacological pretreatment) to alter exosome composition and enhance their therapeutic payload [2,131]. Together, these engineering strategies provide greater precision in modulating specific biological activities and enable more selective therapeutic targeting, resulting in improved specificity, efficacy, and safety of exosome-based therapeutics. A summary of the major engineering strategies applied to stem cell-derived exosomes for wound healing and skin regeneration, along with model systems and key outcomes, is presented in Table 4.

### 4.1. Direct Modification of Exosomes

Direct engineering strategies enhance exosome therapeutic properties by modifying vesicles after isolation through various cargo loading methods, surface modifications, and targeting approaches [2,131]. Major techniques for loading cargo into stem cell-derived exosomes include electroporation, extrusion-based loading, sonication, freeze–thaw cycling, co-incubation, and surface permeabilization treatments [2,131]. Electroporation is one of the most widely used methods for packaging therapeutic cargos into exosomes. In this technique, a high-intensity electric field transiently disrupts the phospholipid bilayer of exosome membranes, creating temporary pores that permit diffusion and uptake of drugs, nucleic acids, or proteins into the exosome interior. After electroporation, the membrane reseals, preserving exosome structure and function and allowing efficient loading of relatively large molecules such as siRNAs or miRNAs [134]. Although electroporation can achieve high loading efficiencies (e.g., up to 31.63%), the application of high voltage pulses may induce membrane damage, vesicle fragmentation, and reduced exosome recovery [135]. This method also typically requires substantial exosome quantities. Consequently, careful optimization of electroporation parameters such as voltage (e.g., 750 V), number of pulses, cargo concentration, and exosome to cargo volume ratio is essential to balance loading efficiency with preservation of exosome integrity and bioactivity [136]. Extrusion-based loading represents another important approach, in which exosomes are co-processed with bioactive substances through an extruder equipped with porous membranes (typically 100–400 nm pore size), mechanically disrupting and reforming membranes to encapsulate cargo [137]. For example, micro extruders with polycarbonate membranes have been used to efficiently incorporate melatonin into exosomes via controlled mechanical extrusion, yielding melatonin-loaded exosomes that enhanced the therapeutic efficacy of topical formulations for atopic dermatitis by improving transdermal delivery and inhibiting mast cell infiltration, local inflammation, and fibrosis [138,139]. Surface permeabilization using agents such as saponin has also been employed to increase exosomal membrane permeability and facilitate loading of small hydrophilic molecules. In addition, sonication (ultrasound treatment) and freeze–thaw cycling have been used to promote cargo entry while maintaining functional exosome preparations for wound healing and skin regeneration [2,131].

Surface modification methods further expand the functional versatility of exosomes. Chemical modification enables precise attachment of therapeutic molecules or targeting ligands to exosome membranes through covalent or non-covalent interactions [140,141]. Conjugated moieties include RGD peptides, transmembrane fusion proteins, polydopamine (PDA) coatings, PEGylation, and antibodies that provide integrin targeting toward fibroblasts and endothelial cells, improved adhesive properties, enhanced colloidal stability, sustained release, prolonged circulation time, reduced immunogenicity, and selective targeting of M1 macrophages for inflammatory modulation [142]. Additional targeting strategies exploit integration of exosomes with functional biomaterials to further enhance therapeutic outcomes by providing supportive delivery depots. Incorporation of exosomes into biocompatible materials such as hydrogels, scaffolds, and nanofibers improves delivery efficiency, stability, and controlled release of therapeutic cargos for wound healing and skin regeneration [51,143,144,145,146,147]. These biomaterial-assisted systems, discussed in more detail in Section 5, enable controlled release at the target site and protect exosomes from premature degradation, features that are particularly important for treating chronic wounds and promoting tissue regeneration. Collectively, direct and biomaterial-integrated engineering strategies have demonstrated markedly improved wound healing with reduced scarring in preclinical models.

### 4.2. Parental Cell Engineering (Preconditioning and Pretreatment Strategies)

Parental MSC engineering strategies include preconditioning and pretreatment-based approaches that modify exosome content at the cellular level. In preconditioning, genetic modification or gene editing tools such as CRISPR/Cas9 are applied to stem cells to alter their genetic material [2]. These modifications can increase exosome yield, enhance surface protein display, enable more precise loading of therapeutic molecules (e.g., specific miRNAs and proteins), and improve targeting of defined pathological processes, thereby promoting exosome uptake by recipient cells without compromising vesicle structural integrity [2,148]. For example, TNF-stimulated gene 6 (TSG 6)-overexpressing MSCs generated via transfection improved wound healing in a mouse model by promoting collagen deposition, reducing local inflammation, and preventing scar formation [149]. Li et al. reported that NRF2-overexpressing ADSCs produced exosomes that effectively promoted angiogenesis and reduced oxidative stress in diabetic wounds [150]. In another study, lentiviral transfection combined with ultracentrifugation-mediated enrichment of miR 132 in ADSCs Exos alleviated inflammation, enhanced angiogenesis, and promoted macrophage polarization toward the M2 phenotype by modulating NF κB signaling, thereby improving wound healing outcomes [151]. Similarly, ADSCs Exos-overexpressing mmu_circ_0001052 activated the FGF4/p38MAPK pathway and improved cell proliferation, migration, and angiogenesis by suppressing apoptosis and miR 106a 5p in diabetic foot ulcers [100]. More broadly, genetically modified ADSCs Exos have been used extensively to treat chronic wounds by selectively enriching exosomal non-coding RNA (ncRNA) content, providing a targeted approach to enhance repair-associated ncRNAs and thereby increase therapeutic efficacy. For instance, Xiang et al. overexpressed miR 21 in ADSCs Exos to promote keratinocyte proliferation, migration, and survival while suppressing apoptosis, thus facilitating re-epithelialization and tissue regeneration [148]. Despite these encouraging results, genetic modification remains technically complex and costly, and raises biosafety concerns; its applicability to other cargo types is also limited, posing significant barriers to broad clinical translation [152].

A second major parental cell engineering approach involves pretreatment or pre-processing of parent cells with pharmacological agents, cytokines, chemical modulators, or specific environmental conditions (such as hypoxia) to transiently enhance exosome yield and therapeutic potency [140,153]. This strategy leverages the intrinsic adaptability of stem cells and is generally simpler to implement than gene editing, making it one of the most commonly employed methods for exosome enhancement [80,82,154]. Chen et al. generated TGF-β1-enhanced exosomes by culturing HucMSCs under hypoxic conditions in the presence of isoproterenol, which improved sweat gland structural and functional restoration and enhanced epidermal tissue repair [57]. Hypoxia-based pretreatment of ADMSCs induced distinct alterations in the miRNA and protein profiles of their exosomes, thereby modulating the therapeutic efficacy of ADMSCs Exos in skin wound repair and regeneration [38,87]. Notably, hypoxia-conditioned ADMSCs Exos (Hpy ADMSCs Exos) showed significant upregulation of wound-associated miRNAs, including miR-126-5p, miR-21-3p, and miR-31-5p, compared with normoxic controls, which potentiated angiogenesis and tissue regeneration through regulation of target signaling pathways [87]. Shi et al. reported that hypoxic preconditioning of ADSCs Exos significantly increased circ Snhg11 expression; exosomal circ Snhg11 was delivered to macrophages, where it modulated polarization via the miR-144-3p/HIF 1α axis and thereby enhanced healing in diabetic foot ulcer models [155]. In a recent study, hypoxia-preconditioned ADSCs Exos (Hpy ADSCs Exos) exhibited superior therapeutic efficacy and improved diabetic wound healing through activation of the PI3K/AKT pathway [38]. Exosomes with anti-inflammatory and pro-angiogenic properties have also been generated by pretreating MSCs with interferon γ (IFN γ) [156], while other pretreatment strategies have been shown to alter the composition and function of ADSCs-derived exosomes [57,140]. Although pretreatment-based strategies are comparatively simple and generally safer than permanent genetic modification, their effects are transient and can vary between batches, leading to inconsistencies in exosome quality and function. Moreover, the limited long-term stability of preconditioned exosomes poses additional challenges for clinical scalability and reproducibility.

**Table 4:** A representative list of engineering strategies of stem cell-derived exosomes for improved wound healing and skin regeneration. The engineering strategies for SC-Exos include both direct and indirect strategies ranging from genetic engineering, hypoxia preconditioning, surface modifications, and integration with biomaterials for targeted delivery.

Exosome Origin	Engineering Strategies	Model	Findings	References
BMMSC-exosomes	Transfection to overexpress lncRNA HOTAIR (HOX transcript antisense RNA)	Diabetic (db/db) mice wound model	Promoted angiogenesis and diabetic wound healing	[156]
HucMSC-exosomes	3D coculture of HucMSCs with endothelial cells under hypoxic conditions (1–5% O_2_)	In vitro study (endothelial cell assays)	Enhanced endothelial cells proliferation and inhibited apoptosis	[115]
miR-29a-modified hADMSCs-exosomes	Transfection of miR-29a mimics into hADMSCs	Thermal injury mouse model	Reduced excessive scar formation and improved healing quality	[157]
TSG-6 modified MSCs exosomes	Lentiviral-mediated TSG-6 overexpression in hBMMSCs	Mouse full-thickness wound model	Suppressed scar formation; reduced inflammation and collagen deposition	[148]
BMSC-exosomes	Electroporation-mediated loading of miR-542-3p	Mouse skin wound model	Facilitated cellular proliferation, enhanced collagen deposition, stimulated neovascularization, and accelerated rapid wound closure	[158]
ADMSCs-exosomes	Hypoxic preconditioning (1% O_2_, 48 h)	Rat full-thickness skin injury model	Accelerated wound closure; improved collagen deposition and angiogenesis	[97]
ADMSCs-exosomes	Genetic engineering (lentiviral miR-21 overexpression)	Full-thickness dermal wound in BALb/c mouse	Enhanced migration and proliferation of HaCaT cells, improved wound healing	[159]
ADMSCs-exosomes	Selenium preconditioning (Se-treatment of ADMSCs)	Diabetic (db/db) mice wound model	Superior wound healing, reduction of oxidative stress, enhanced tissue generation	[160]
ADMSCs-exosomes	Hypoxic preconditioning (1–5% O_2_)	Immunodeficient mice wound model	Enhanced wound healing efficacy compared to normoxic ADMSC-Exos	[161]
ADMSCs-exosomes	Oxygen nanobubble coating + hydrogel integration	Male rat full-thickness wound model	Enhanced wound healing; mitigated hypoxia; inhibited inflammation; promoted angiogenesis; facilitated sustained exosome delivery	[162]
ADMSCs-exosomes	Hypoxic pretreatment + hydrogel-based delivery	Diabetic wound	Increased migration, proliferation, and angiogenesis of vascular endothelial cells; accelerated diabetic wound healing via circRNA delivery	[163]
ADMSCs-exosomes	FGF1 preconditioning (treatment of ADMSCs with FGF1)	Rat ischemic skin flap model	Enhanced cell viability; inhibited oxidative stress; alleviated apoptosis and pyroptosis; significantly improved survival rate of ischemic skin flaps	[164]

Abbreviations: BMMSCs—bone marrow mesenchymal stem cells, ADMSCs—adipose derived mesenchymal stem cells, FGF1—fibroblast growth factor 1, miR—micro-RNA, TSG-6—tumor necrosis factor (TNF)-stimulated gene/protein-6, HOX—homeobox gen5., lncRNA—long non-coding RNA. HOTAIR—HOX transcript antisense RNA (“h” before the abbreviations stand for human derived).

## 5. Biomaterial-Assisted Exosome Delivery Systems

The development of effective and advanced delivery systems for stem cell-derived exosomes is critical to overcome the limitations of free exosome administration, such as rapid clearance, poor bioavailability, and loss of therapeutic efficacy, while preserving exosome structural integrity and biological activity [51,143,144,145,146,147]. In addition, optimized delivery platforms enable more precise control over inflammatory resolution, angiogenesis, and tissue remodeling in both acute and chronic wounds. This section discusses engineering strategies that combine biomaterials with exosomes as delivery systems for skin wound repair and regeneration. Traditionally, stem cell-derived exosomes have been administered via intravenous or subcutaneous injection to improve wound healing and skin regeneration. Although these routes can stimulate fibroblast activity and promote macrophage polarization to accelerate wound closure, their invasive nature and the rapid systemic clearance of exosomes from the wound site substantially limit therapeutic efficacy [165,166,167].

**Figure 6:**
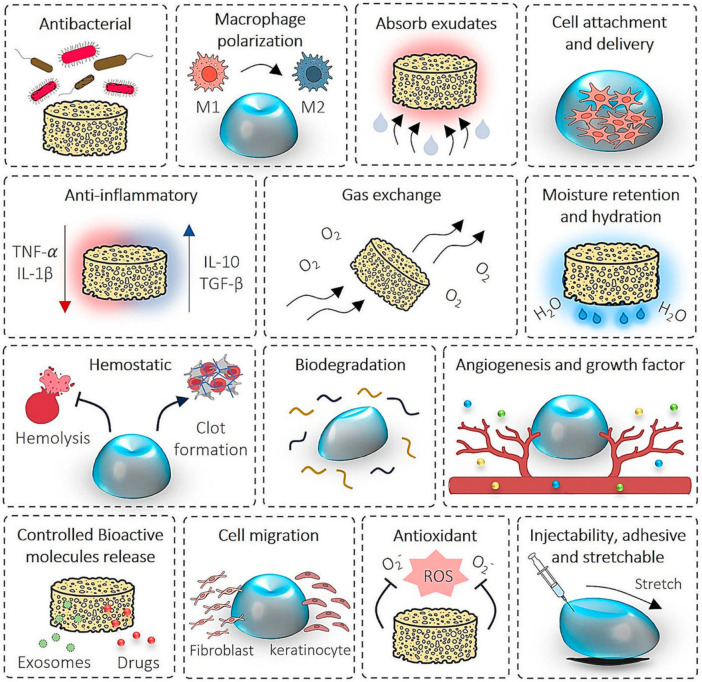
This schematic depicts comparative structural, functional, and biomedical attributes of various biomimetic biomaterials integrated with stem cell-derived exosomes for enhanced wound healing, specifically illustrating porous scaffold and hydrogel scaffold as representative models. Notably, key characteristics depicted such as porosity, mechanical strength, biocompatibility, cell adhesion, and support for vascularization are shared with other advanced scaffold designs, including nanofiber scaffolds and three-dimensional printed scaffolds. The illustration emphasizes overlapping and unique properties among these platforms, highlighting their respective suitability for cell proliferation, differentiation, and integration within regenerative medicine applications. Reproduced from [171] with permission from BMC. This work is licensed under a Creative Commons Attribution 4.0 (CC BY) International License.

To address these shortcomings, current delivery strategies are increasingly focused on advanced biomaterial-based systems—such as hydrogels, porous and 3D-printed scaffolds, and nanofibers—to achieve sustained, localized, and controlled release of stem cell-derived exosomes while enhancing stability and therapeutic potency [67,140]. The flexible morphologies and tunable physicochemical properties of these biomaterials allow the generation of diverse exosome-loaded constructs tailored to promote wound healing and skin regeneration [168,169]. Among these, integration of stem cell-derived exosomes with hydrogels has emerged as a particularly promising approach to enhance stability, prolong residence time, and improve therapeutic outcomes in wound healing and skin regeneration without compromising exosome bioactivity (Figure 6) [170]. A representative list of biomaterial-based delivery systems for the release of stem cell-derived exosomes to wound sites is presented in Table 5.

Hydrogels—three-dimensional, highly hydrophilic biomaterials—stand out as advanced delivery vehicles for stem cell-derived exosomes [67,172]. They address limitations related to exosome stability, retention, and control over release kinetics, enabling sustained exosome delivery with significantly improved therapeutic performance by tailoring release profiles to the wound microenvironment [173,174,175]. Hydrogels derived from both natural and synthetic polymers are frequently integrated with exosomes using diverse fabrication techniques, including dropwise addition, co-cultivation, and freeze-drying, to optimize exosome stability, bioactivity, and controlled-release characteristics for therapeutic applications [130,145,147,167,176]. Multiple hydrogel-based loading strategies have been developed: (1) direct loading of exosomes by exploiting hydrogel swelling capacity and pore size; (2) blending exosomes with hydrogel precursor solutions via physical interactions or chemical crosslinkers; and (3) in situ gelation using double syringe systems for localized, minimally invasive administration and controlled release [56,167]. These strategies offer high tunability, along with adjustable mechanical strength and degradation rates; however, challenges such as excessively large pore sizes and the need for potentially cytotoxic crosslinkers can compromise precise exosome release. Hydrogel scaffolds prepared from gelatin methacryloyl (GelMA), silk fibroin, hyaluronic acid, chitosan, and collagen enhance wound repair by promoting angiogenesis, re-epithelialization, and ECM remodeling through controlled, sustained release of stem cell-derived exosomes over extended periods [70,177,178]. Furthermore, 3D printed hydrogel constructs facilitate wound healing and skin regeneration by enabling precise spatial and temporal distribution of exosomes, thereby optimizing local therapeutic delivery and cell–matrix interactions [179].

Several studies have demonstrated the benefits of hydrogel-based exosome delivery in preclinical wound models. BMMSCs Exos and ADSCs Exos incorporated into collagen, alginate, Pluronic F 127, or multifunctional polysaccharide-based hydrogels (FEP) promoted wound healing via sustained exosome release at the injury site, enhanced collagen regeneration, increased neovascularization, and reduced inflammation [69,169,178,180]. In another report, epidermal stem cell-derived exosomes loaded into VH298-containing GelMA hydrogels improved blood supply and angiogenesis in a mouse diabetic ulcer model by enhancing HUVEC function through activation of the HIF-1α pathway [130]. A thermosensitive hydrogel composed of diethylene glycol monomethyl ether methacrylate (DEGMA) and hyaluronic acid (HA), loaded with MSC-derived exosomes enriched in miR-24-3p, promoted epithelial cell migration and reduced inflammation and fibrosis [43]. Platelet-rich plasma (PRP) gels combined with MSC Exos have also shown promise for wound treatment [181]. Bakadia et al. developed bioengineered microporous three-dimensional amniotic membrane scaffolds and silk protein-based double crosslinked bioactive hydrogel dressings (SP Exos) as advanced exosome delivery platforms, which enhanced the rate of diabetic wound healing by supporting sustained release of bioactive cargos, stimulating angiogenesis and ECM remodeling, and providing antimicrobial effects in in vivo full-thickness diabetic ulcer models [182]. Sustained exosome release from hydrogels promotes granulation tissue formation, intensifies collagen deposition, shifts macrophage polarization toward the M2 phenotype, facilitates neovascularization, regenerates sebaceous glands and hair follicles, and attenuates NETosis and oxidative stress.

Beyond hydrogels, a range of porous and nanofibrous scaffolds, including biomimetic 3D printed matrices, have been extensively used as exosome delivery platforms to achieve sustained release, protect exosomal integrity, and support cellular infiltration, thereby facilitating wound healing and skin regeneration through improved angiogenesis, ECM remodeling, and immunomodulation [179,183]. In one study, a multifunctional bioscaffold embedded with ADSC Exos exhibited antimicrobial, oxygen-releasing, and antioxidant properties that accelerated the resolution of diabetic wounds complicated by Staphylococcus aureus and Pseudomonas aeruginosa infection, via enhanced antimicrobial activity and modulation of the local inflammatory milieu [184]. Integration of such scaffolds with ADSC Exos produced synergistic effects by boosting collagen remodeling and angiogenesis while reducing oxidative stress. Similarly, implantation of ADSC Exos-loaded bioengineered microporous three-dimensional amniotic membrane scaffolds (AMS) significantly improved diabetic wound healing by robustly stimulating angiogenesis and vascular remodeling within the wound microenvironment [49].

Nanofiber-based delivery systems further leverage high surface-area-to-volume ratios, ECM-mimicking nanoscale architectures, and tunable porosity to enhance exosome stability and bioavailability during wound healing and skin regeneration [185]. Qian et al. fabricated HucMSC-derived exosome-loaded chitosan–silk fibroin–silver dressings that promoted human fibroblast proliferation, collagen matrix synthesis, neovascularization, and neural regeneration while providing strong antibacterial effects in vitro and in a Pseudomonas aeruginosa–infected murine full-thickness wound model [181]. In another report, electrospun polycaprolactone (PCL) fibers incorporating BMMSCs Exos facilitated wound healing by promoting beneficial macrophage and regulatory T cell responses in skin wounds [186,187]. More recent studies have shown that nanofibers fabricated from gelatin, silk fibroin, PCL, and composite materials effectively incorporate exosomes and support controlled delivery over extended periods while maintaining exosome bioactivity at wound sites [143,144]. Loading strategies such as surface adsorption and encapsulation enable sustained cargo release, promoting re-epithelialization, collagen deposition, angiogenesis, and rapid wound closure in acute and chronic (particularly diabetic) wounds with reduced scarring [47,143,144]. Mesenchymal stem cell-derived exosome-loaded nanofiber scaffolds thus modulate the wound microenvironment and key signaling pathways and represent promising next-generation therapeutic platforms with improved healing outcomes.

Despite these advances, clinical translation of biomaterial-assisted exosome delivery systems for skin wound repair remains hampered by several challenges. These include incomplete characterization of exosomal cargos (microRNAs, mRNAs, proteins, and lipids), heterogeneity across cell sources, limited understanding of exosome-mediated intercellular communication, and discrepancies between in vitro and in vivo release profiles. Addressing these issues will require a deeper investigation of exosomal composition, intrinsic targeting capabilities, biological functions, and pharmacokinetic and pharmacodynamic properties to enable safe, reproducible, and effective therapeutic applications in human wound care.

**Table 5:** Different delivery systems utilized for delivery of stem cell-derived exosomes for wound healing and skin regeneration.

Source of Exosomes	Delivery System/Biomaterials Utilized	Mechanism	Findings	References
ADMSCs	Hydrogel/Alginate	Sustained release promoted angiogenesis and collagen synthesis	Enhanced re-epithelialization, increased collagen synthesis, and improved wound closure (83.6%),	[178]
UCMSCs	Hydrogel/Silk fibroin/silk sericin	Mechanical support with controlled exosome release, angiogenesis promotion	Promoted wound healing and angiogenesis, enhanced tissue regeneration	[188]
MSCs	Hydrogel/HA	Injectable delivery, promotes osteogenic and angiogenic pathways	Promoted osteogenesis and angiogenesis, upregulated ALP, OCN, COL1A1 expression	[189]
UCMSCs	Hydrogel/Gelatin	Direct loading and gradual release, hemostatic properties	Superior hemostasis, increased collagen fiber formation, enhanced angiogenesis (CD31^+^)	[72]
ADMSCs	Hydrogel/GelMA	Sustained release, mechanical activation of signal transduction pathways such as TGF-β/Smad signaling pathway	Enhanced fibroblast proliferation, inhibited excessive collagen fiber deposition and the overexpression of the angiogenic factor VEGF.	[144]
UCMSCs	Hydrogel/dECM	Hypoxia induction, activation of classical HIF-1α signaling pathway.	Promoted endothelial cell proliferation, migration, and tube formation, inhibition of SERPINE1 activity in endothelial cell	[51]
ADMSCs	Hydrogel/Chitosan-PEG	Controlled release, activation of NF-κB pathway	Enhanced endothelial cell migration, reduced oxidative stress, and promoted angiogenesis	[145]
MSCs	Hydrogel/HA	Sustained release, extracellular matrix remodeling, upregulation of VEGF	Enhanced proliferation and migration of endothelial cells and fibroblasts, enhanced angiogenesis, promotion of re-epithelialization, and accelerated wound closure	[146]
BMMSCs	Scaffold/decellularized ECM	Native ECM architecture retention, growth factor preservation	Facilitated cellular infiltration, tissue regeneration, vascular network formation	[190]
DPMSCs	Scaffold/Collagen	Up-regulation of miRNAs (hsa-miR-21-5p and hsa-miR-29a-5p)	Enhanced migration and proliferation of HDFs, angiogenesis promotion in HUVECs	[191]
UCMSCs	Scaffold/collagen	Inducing of anti-inflammatory M2-macrophage polarization	Promoted cell migration and proliferation at wound sites, accelerated vascular remodeling, re-epithelialization, and wound closure	[61]
MSCs	Nanofibers/PCL	Surface adsorption and controlled release, ECM-mimicking structure	Mitigated reactive astrocytes/microglia, elevated growth-associated proteins	[192]
ADMSCs	Nanofibers/PCL-gelatin	Encapsulation within fiber matrix, sustained local delivery	Enhanced wound closure, improved collagen formation, reduced inflammation	[143]

Abbreviations: ADMSCs—adipose derived mesenchymal stem cells, DPMSCs—dental pulp derived mesenchymal stem cells, UMSCs—urine derived mesenchymal stem cells, GMSCs—gingival derived mesenchymal stem cells, UCMSCs—umbilical cord derived stem cells, miR—micro RNA, ECM—extra cellular matrix, PEG—poly ethylene glycol, ALP—alkaline phosphatase, OCN—osteocalcin, col1A1—collagen I, PCL—poly caprolactone, HA—hyaluronic acid, GelMA—gelatin methacryloyl, HDF—human dermal fibroblasts, HUVECs—human umbilical vein endothelial cells, VEGF—vascular endothelial growth factor, HIF—hypoxia inducing factor, TGF—transforming growth factor, NF-κB—Nuclear factor kappa-light-chain-enhancer of activated B cells, SERPINE1—Serine Protease Inhibitor Clade E Member 1.

## 6. Clinical Trials and Updates

Stem cell-derived exosomes present a transformative cell-free therapeutic strategy, demonstrating substantial efficacy across diverse preclinical models of wound healing and skin regeneration [11,192]. In recent years, clinical translation has progressed from early-phase safety studies to more advanced efficacy-oriented trials; however, the overall number of well-characterized clinical studies remains limited relative to the extensive preclinical evidence [11,166,192,193]. A representative list of clinical trials of SC-Exos for wound healing and skin regeneration, with their updated status and outcomes, is presented in Table 6. Despite promising preclinical safety data, the clinical utility of SC-Exos remains in its early stages, with several phase I/II trials investigating safety and preliminary efficacy in chronic wounds. While preclinical models demonstrate favorable safety profiles, comprehensive clinical safety data—including long-term outcomes and rare adverse events—are still required.

The most robust data to date originate from a single-center randomized controlled clinical trial evaluating Wharton’s jelly mesenchymal stem cell-derived exosomes (WJ-MSC-Exos) in patients with diabetic foot ulcers (DFUs) [194]. In this phase I study (NCT04134676), 110 adults with chronic DFUs were randomized to receive weekly topical WJ-MSC-Exos plus standard of care (SOC), SOC alone, or SOC plus carboxymethyl cellulose vehicle for 4 weeks [194]. The primary endpoints included the proportion of ulcers achieving complete closure and time to full epithelialization, while safety was assessed based on the frequency of adverse events. Overall, 53 patients (62%) achieved complete healing, but the exosome-treated group showed a significantly higher proportion of fully healed ulcers and a markedly shorter mean time to complete epithelialization (approximately 6 weeks, range 4–8 weeks) compared with control groups, which required around 20 weeks (range 12–28 weeks) [194]. These findings indicate a clinically meaningful acceleration of wound closure when WJ-MSC-Exos are added to SOC. No serious treatment-related adverse events were reported, and WJ-MSC-Exos were considered safe and well tolerated, although the available report provides limited quantitative detail on local and systemic adverse events, restricting comprehensive risk characterization.

Clinical development of exosome-based products has accelerated further, with several trials evaluating different stem cell-derived exosome formulations for wound healing and skin regeneration over the past six years [11,166,192,193]. These include trials of bone marrow MSC exosomes in diabetic cutaneous ulcers (NCT05243368), adipose tissue MSC exosomes delivered via hydrogel dressings in chronic wounds (NCT05475418), and umbilical cord Wharton’s jelly MSC-derived exosomes formulated as a topical gel for chronic DFUs (NCT06812637), in which once-weekly application for 4 weeks is being investigated with percent wound closure, ulcer healing rates at 20 weeks, and scar/tissue-quality assessments as primary or key secondary endpoints. However, detailed results on population-level outcomes and adverse events from these ongoing studies have not yet been fully published, and their contributions currently lie in establishing feasibility and informing future trial design.

In addition, platelet-derived exosome formulations have entered clinical testing. Purified Exosome Product (PEP), a freeze-dried platelet exosome powder reconstituted with fibrin sealant, is being evaluated in a phase 2a clinical study in patients with DFUs following an earlier phase 1B Mayo Clinic trial that primarily established feasibility and short-term safety [195]. The phase 2a study is designed to assess both safety and efficacy (e.g., percentage wound closure and time to healing), but detailed outcome data and adverse-event profiles have not yet been reported in peer-reviewed form. Given the potent pro-healing but also potentially pro-coagulant and pro-inflammatory cargos of platelet-derived exosomes, careful dose-finding, rigorous safety monitoring, and long-term follow-up will be essential [193,195].

Beyond DFUs, several early-phase dermatologic studies are evaluating MSC-derived exosomes in conditions such as psoriasis, photoaging, alopecia, and dystrophic epidermolysis bullosa, generally enrolling small patient populations (often fewer than 50 participants) and focusing primarily on safety, tolerability, and exploratory efficacy endpoints [11,166,192,193]. Preliminary reports suggest acceptable short-term safety and improvements in surrogate clinical parameters (e.g., lesion scores, skin elasticity and hydration); however, most studies are single-center, underpowered, and lack standardized primary endpoints or detailed adverse-event reporting, which limits direct comparison and meta-analytic synthesis. Collectively, current clinical data support the feasibility and short-term safety of stem cell-derived exosome therapies and provide encouraging signals of efficacy in chronic and complex wounds, but they also highlight the need for larger, multicenter randomized controlled trials with harmonized outcome measures and comprehensive safety evaluation to fully define the risk–benefit profile and guide regulatory approval [11,166,192,193,194,195]. Current clinical trials suggest that stem cell-derived exosome products can be administered topically or locally with acceptable short-term safety in small cohorts, consistent with preclinical findings. However, most studies are single-center and underpowered, with limited reporting of detailed adverse events and no long-term follow-up data, underscoring the need for larger randomized controlled trials to fully characterize the safety profile.

## 7. Challenges and Future Directions

Despite notable advances in mechanistic understanding, delivery science, scalable manufacturing, and early clinical evaluation of stem cell-derived exosomes, several critical barriers must be addressed before these products can be widely implemented in routine wound care. Key challenges span scientific and technical issues, manufacturing and quality control, regulatory and ethical considerations, and clinical trial design and translation [196]. From a scientific and technical standpoint, product heterogeneity remains a major obstacle. Exosomes are isolated from diverse stem cell sources using different methods (e.g., ultracentrifugation, precipitation, immunoaffinity, size-based or microfluidic techniques) and are formulated in multiple dosage forms (liquid suspensions, lyophilized powders, hydrogels, and scaffolds), resulting in substantial variability in cargo composition, purity, and bioactivity that complicates dose standardization and mechanistic attribution. Identifying robust potency assays is particularly challenging because exosomes are complex, multi-component systems, and no single biomarker or functional readout yet reliably predicts clinical benefit across indications [41]. Stability and formulation present additional constraints: exosomes are sensitive to storage conditions, repeated freeze–thaw cycles, high shear, and certain solvents, all of which can alter vesicle integrity and biological activity. Although lyophilization and other stabilization approaches are being explored to improve shelf life and handling, these strategies introduce new variables that must be tightly controlled. Poor retention at the wound bed is another practical challenge; integration with biomaterial carriers such as hydrogels, scaffolds, and nanofibers improves local retention and protects exosomes from rapid clearance but also adds complexity to manufacturing, characterization, and regulatory review [197]. In addition, identifying a single predictive potency is challenging, as exosomes are complex, multi-component systems. This lack of identifying the predictive potency of SC-Exos would make it difficult to translate into clinical settings.

Scaling up production while maintaining batch-to-batch consistency is a further hurdle. Large-scale culture, isolation, and purification must be implemented under good manufacturing practice (GMP) conditions, with rigorous quality control to minimize co-isolated contaminants and ensure reproducible cargo profiles. Safety considerations remain a priority, including potential residual host cell DNA, viral particles, unintended pro-fibrotic or tumor-promoting signals, and immunogenic responses influenced by the exosome source and route of administration. While preclinical studies show low immunogenicity, clinical trials must include rigorous biodistribution studies and long-term safety monitoring to confirm these findings [197,198,199]. Regulatory frameworks are still evolving; major agencies such as the FDA and EMA currently classify therapeutic exosomes as biological products, but clear, harmonized guidelines specific to exosome-based medicines remain limited. This lack of standardized classification and expectations regarding starting materials, potency criteria, manufacturing controls, sterility, and clinical evidence can slow development and approval pathways. In parallel, ethical and public health concerns are amplified by the rapid commercialization and cosmetic marketing of poorly characterized “exosome” formulations, prompting regulators and professional societies to issue guidance and enforcement actions against unlicensed or unproven clinical use.

Clinical and translational bottlenecks include heterogeneous trial designs, variability in endpoints, and high costs associated with GMP biomanufacturing and quality control. Clinically meaningful endpoints—such as time to complete closure, percentage closure at predefined time points, infection and reintervention rates, limb salvage in diabetic foot ulcers, and validated scar quality scores—are not yet standardized across studies, limiting the ability to compare outcomes and perform meta-analyses. Future trials will need to be larger, adequately powered randomized controlled studies with harmonized outcome measures and appropriate comparators to definitively demonstrate superiority over standard of care [196]. Looking forward, progress will likely depend on coordinated efforts across several fronts. Technological priorities include the development of advanced, GMP-compliant, scalable manufacturing platforms; robust, mechanism-linked potency assays; and better analytical tools to profile exosomal microRNAs, mRNAs, proteins, lipids, and functional properties. On the delivery side, smart, stimuli-responsive biomaterials—capable of releasing exosomes in response to local cues such as pH, reactive oxygen species, or enzymatic activity in chronic wounds—could further improve on-site retention and reduce dosing frequency. In parallel, emergent bioengineering strategies such as rational cargo loading (miRNAs, siRNAs, and proteins), genetic modification of parental cells, and surface engineering of exosomes with peptides or antibodies are poised to enhance targeting precision and functional potency, enabling more tailored modulation of the wound microenvironment. Equally important will be strong regulatory and ethical oversight, along with stakeholder engagement involving clinicians, scientists, regulators, and patient communities to align expectations and address commercialization challenges. With concerted advances in manufacturing, delivery, regulatory science, and clinical trial methodology, stem cell-derived exosomes could ultimately transition from experimental biologics to standardized, evidence-based therapeutics for complex skin injuries and chronic wounds. Recent advances in artificial intelligence, spanning drug discovery and pharmaceutical development, as well as clinical decision support and predictive modeling in oncology, highlight the potential of artificial intelligence-driven frameworks to accelerate exosome engineering and guide the design of next-generation wound healing therapeutics [200,201].

## 8. Conclusions

Stem cell-derived exosomes have emerged as an exciting and innovative cell-free therapeutic avenue for wound healing and skin regeneration, with profound therapeutic implications. SC-Exos from various stem cell sources, such as MSCs, iPSCs, and others, bypass many of the limitations often associated with whole-cell transplantation, including low survival rates, heterogeneity, and safety concerns about tumorigenicity. The SC-Exos cargo, rich in proteins, lipids, and regulatory RNAs, orchestrates multiple processes essential for wound healing, including modulation of inflammatory responses, stimulation of fibroblast activity, enhancement of angiogenesis, and promotion of extracellular matrix remodeling. Additionally, direct and indirect exosome engineering strategies, such as genetic editing, pretreatment, and targeting strategies (integration with biomaterials), accelerate wound repair by modulating different phases of the wound healing process. SC-Exos integrated with biomaterials, such as hydrogels, scaffolds, and nanofibers, have greatly enhanced skin wound repair and regeneration in various preclinical studies. However, challenges remain in standardizing isolation methods and addressing biological, manufacturing, and regulatory issues to fully realize their clinical potential. To address these concerns, efforts have focused on standardization, engineering strategies, targeted delivery, and large-scale clinical validation to enable successful translation. With the integration of bioengineering, biomaterial platforms for targeted delivery, clinical research, translational advancements in SC-Exos, and regulatory science, this therapeutic approach holds great promise to become a safe, effective, and personalized treatment for complex skin injuries and chronic wounds in the near future. Taken together, stem cell-derived exosomes enriched with diverse cargo present a paradigm shift in regenerative medicine for wound healing and skin tissue engineering.

## Figures and Tables

**Table 1:** A representative isolation method of SC-Exos showing comparative analysis in terms of purity, yield, scalability, cost, therapeutic function and limitations for wound healing applications.

Method	Yield	Purity	Scalability/Cost	Therapeutic Outcomes	Limitations/References
Differential ultracentrifugation	Low–moderate (10^10^–10^11^ particles/mL)	High (minimal protein contamination)	Poor (low throughput)/High (requires ultracentrifuge)	Preserves bioactivity; standard reference method	Time-consuming (16–24 h); low yield; potential cargo damage from high g-forces [45,46]
Size-exclusion chromatography (SEC)	Moderate–high	Very high (size-based separation)	Moderate (column capacity limits)/Moderate	Excellent cargo integrity; minimal aggregation; superior for wound healing studies	Moderate yield; requires pre-concentration; column fouling [45]
Precipitation (e.g., PEG, polymer-based)	High (up to 10^12^ particles/mL)	Moderate (protein co-precipitation)	Good/low	Rapid; scalable for clinical use	Lower purity; potential chemical residues; variable bioactivity [45]
Ultrafiltration	High	Moderate	Excellent/Low	High throughput; GMP-compatible	Membrane clogging; potential shear damage [45,46]
Immunoaffinity capture	Low–moderate	Highest (marker-specific)	Poor/Very high	Highly specific; ideal for functional studies	Expensive; marker bias; low yield [44,45]
Microfluidic/acoustic (emerging)	Moderate–high	High	Excellent (continuous flow)/High (initial setup)	Scalable; gentle; GMP potential; promising for clinical manufacturing	Emerging; limited validation [44,45]

**Table 2:** Comparative mechanisms of BMMSC-, ADMSC-, and HucMSC-derived exosomes in wound healing: key functional aspects, targets, cargo signatures, and therapeutic profiles.

Mechanistic/Functional Aspect	BMMSCs-Derived Exosomes (BMMSCs-Exos)	ADMSCs-Derived Exosomes (ADMSCs-Exos)	HucMSCs-Derived Exosomes (HucMSCs-Exos)
Source	Bone marrow aspirates	Subcutaneous adipose (liposuction)	Wharton’s jelly, umbilical cord
Targets	Dermal fibroblasts and endothelial cells (ECs)	Fibroblasts, keratinocytes, ECs	Fibroblasts, keratinocytes, ECs
Key cargo and molecular signatures	Integrin-binding proteins anti-ferroptosis RNAs [44]	Cell adhesion proteins, anti-fibrotic miRNAs/lncRNAs [44]	VEGF pathways, AKT/ERK/STAT3/β-catenin [44]
Angiogenesis and vascular remodeling	Wnt/β-catenin, Nrf2; strong in diabetic wounds [44,64]	Moderate perfusion improvement [65]	Strongest CD31^+^ vessels, stable networks [44]
Modulation of inflammation and oxidative stress	↓ Ferroptosis, chronic inflammation [66]	↓ Pro-inflammatory cytokines (UV/senescent skin) [44]	↓ Oxidative stress, pro-healing shift [67]
Re-epithelialization	Indirect via fibroblasts/ECs [61]	Superior keratinocyte proliferation/migration [61]	Rapid with strong angiogenesis support [68]
ECM remodeling and scar modulation	↓ TGF-β/Smad fibrosis [66]	↑ α-SMA/FGF2/collagen III/I balance [44,69]	Improved collagen organization, ↓ scarring [70]
Advantages/limitations	Well-characterized; invasive harvest, lower yield	High yield, minimally invasive	High yield, allogeneic; perinatal sourcing

**Table 3:** A representative list of exosomes derived from different stem cell sources for wound healing and skin regeneration. Stem cell-derived exosomes target diverse cells, ranging from fibroblasts, keratinocytes, HUVECs, immune cells, and other wound-associated cells, using various mechanisms by activating/downregulating different signaling pathways for improved wound healing along with regeneration of cutaneous skin appendages (hair follicles and sebaceous glands).

Source of Exosomes	Target Cells/Study or Model Type	Mechanism	Findings	References
BMMSCs	Fibroblasts, endothelial cells/in vivo	Activation of the Nrf2 pathway	Enhanced diabetic foot ulcer wound healing through decreased ferroptosis, improved cellular viability and function, increased angiogenesis, and reduced oxidative tissue damage	[66]
BMMSCs	Endothelial cells/in vivo	Enhances the expression of miR-383/VEGFA axis and activation of the Wnt/β-catenin signaling pathway	Promotion of angiogenesis, improved vascularization, and hastened wound closure in diabetic cutaneous wounds	[67]
BMMSCs	HaCaT cells, HDFs/in vivo	Downregulation of TGF-β1, Smad2, Smad3, Smad4; upregulation of TGF-β3, Smad7	Promoted proliferation of both keratinocytes and fibroblasts, reduced scar formation, accelerated cutaneous wound healing in rat full-thickness wound model	[68]
ADMSCs	HDFs, keratinocytes, endothelial cells/in vivo	Promotion of cell proliferation, migration, and extracellular matrix remodeling	Increased wound closure rates, promoted fibroblast proliferation, migration, and ECM gene expression (α-SMA, FGF2, elastin, collagen), increased vascularization and tissue regeneration, enhanced re-epithelialization and collagen III deposition in porcine wound model	[69]
ADMSCs	Mouse fibroblasts, cutaneous wound cells/in vivo	Promotion of proliferation and migration of fibroblasts, activation of wound healing-related pathways	Enhanced re-epithelialization and increased collagen expression, significantly rapid wound closure rate in rat skin injury model	[70]
ADMSCs	HDFs, particularly UV-induced senescent fibroblasts implicated in skin photoaging/in vitro	Reduction of senescence markers (p16, p21) and senescence-associated β-galactosidase activity in UV-damaged HDFs	Improved morphology and proliferation of aged fibroblasts, reduced wrinkles and skin thickness, increased collagen density, improved elasticity and hydration, and decreased expression of MMPs associated with collagen breakdown.	[42]
HucMSCs	HUVECs, diabetic wound endothelial cells/in vivo	Amelioration of oxidative stress and promotion of angiogenesis	Accelerated diabetic cutaneous wound healing in vivo, improved oxidative stress injury, enhanced angiogenesis (increased CD31^+^ cells) in diabetic wounds; promoted re-epithelialization, collagen deposition, and ECM remodeling	[71]
HucMSCs	Dermal fibroblasts, endothelial cells, keratinocytes/ in vivo	Activation of AKT, ERK, STAT3, and β-catenin signaling pathways, blocking the TGF-β/Smad2 pathway	Promoted fibroblast proliferation, migration, and collagen synthesis (Types I and III), stimulated angiogenesis, reduction of pro-inflammatory cytokines, rapid wound closure, improved collagen organization, enhanced elastic fiber formation, and reduced scarring	[60]
HucMSCs	Wound site cells, vascular endothelial cells/in vivo	Promotion of cell proliferation and angiogenesis	Superior hemostatic effect, significantly accelerated full-thickness skin wound healing, increased collagen fiber synthesis, CD31^+^ blood vessel formation, and Ki67^+^ cell proliferation, promoted regeneration of hair follicles and sebaceous glands	[72]
PMSCs	Fibroblasts, endothelial cells, keratinocytes, and immune cells/in vivo	Modulation of the inflammatory microenvironment, ECM remodeling	Increased wound closure rate and shortened healing time in chronic burn wounds, promoted greater vascularization increased fibroblast proliferation, collagen fiber deposition, and neoangiogenesis in wound areas	[48]
PMSCs	Cutaneous fibroblasts (EN1^+^ fibroblast lineage), cutaneous appendage cells, vascular endothelial cells/in vivo	Downregulation of YAP signaling pathway leading to inhibition of (EN1 activation	Accelerated wound healing rate and improved healing quality, decreased collagen I/collagen III ratio (anti-scarring effect), enhanced regeneration of hair follicles and sebaceous glands significantly reduced EN1^+^ fibroblast activation (scar-forming cells)	[73]
DPMSCs	PDLSCs, macrophages (RAW264.7 cells)	Inhibition of IL-6/JAK2/STAT3 signaling pathway, macrophage polarization from M1 to M2 phenotype	Promoted proliferation, migration, and osteogenic differentiation of PDLSCs; reduced inflammation, enhanced osteogenesis, reduced alveolar bone loss and promoted periodontal tissue repair in rat periodontitis model	[74]
DPMSCs	HUVECs, cutaneous wound cells/in vivo	Activation of Cdc42/p38 MAPK signaling pathway	Accelerated cutaneous wound healing by promoting neovascularization, enhanced migration, proliferation, and capillary tube formation of HUVECs	[75]
DPMSCs	Multiple regenerative target cells in various tissues/in vitro and in vivo	Regulation of anti-inflammatory pathways	Stimulated regenerative mechanisms, modulated immune responses	[76]
iPSCs	Cutaneous wound cells, immune cells/in vivo	Immune evasion, enhancing cellular migration and proliferation, paracrine signaling	Accelerated skin wound healing (wound closure, epithelialization, collagen deposition, angiogenesis), better immune tolerance with reduced T lymphocyte infiltration	[77]
iPSCs	Fibroblasts, keratinocytes, endothelial cells, immune cells, neural cells/in vitro and in vivo	Stimulated angiogenesis via Notch/VEGFR2 pathway, protect liver tissue via S1P/sphingosine kinase signaling, and maintain pluripotency signaling	Enhanced wound healing with superior efficacy in promoting keratinocyte proliferation, migration, promoted nerve regeneration and functional recovery	[44]
iPSCs	HaCaT cells, cutaneous wound cells/in vitro and in vivo	FGF2-mediated p38 pathway activation, hiPSC exosomes contain FGF-2 that targets FGFR3 to activate p38 signaling	Promoted skin wound healing by inhibiting inflammation (IL-1β, Ccl2, Cxcl5, Ccl7) and enhancing proliferation (increased PCNA^+^ cells)	[78]
EpSCs	Vascular endothelial cells at diabetic wounds/in vivo	Alleviation of excessive autophagy-induced endothelial cell apoptosis	Improved endothelial cell viability and function in diabetic wound microenvironment, promoted diabetic wound healing by protecting vascular endothelial cells from oxidative stress and autophagy-induced apoptosis	[79]
EpSCs	HSFBs/in vitro	Phosphorylation of PKN1 and cyclin expression	Enhanced wound healing, increased collagen synthesis with favorable collagen III/I ratio, dose-dependent effects	[80]
EpSCs	Skin cells in wound tissue, fibroblasts, keratinocytes/in vivo	Downregulation of TGF-β1 in wound healing; promotion of skin regeneration and reduced scarring	Increased wound healing rate and reduced scar formation in rat full-thickness wound model, enhanced angiogenesis and collagen deposition with favorable ECM composition, improved regeneration of cutaneous appendages (hair follicles, sebaceous glands)	[81]

Abbreviations: BMMSCs—bone marrow mesenchymal stem cells, DPMSCs—dental pulp mesenchymal stem cells, PMSCs—placental mesenchymal stem cells, EpSCs—epidermal stem cells, ADMSCs—adipose derived mesenchymal stem cells, HUVECs—human umbilical vein endothelial cells, HaCaT—human keratinocytes cells, HDFs—human dermal fibroblasts, Nrf2—nuclear factor erythroid 2-related factor 2, HSFBs—human skin fibroblasts, HucMSCs—human umbilical cord mesenchymal stem cells, PDLSCs—periodontal ligament stem cells, EN1—Engrailed-1, ECM—extracellular matrix, MAPK—mitogen-activated protein kinase, TGF—transforming growth factor, PKN1—protein kinase N1, YAP—yes-associated protein, FGF—fibroblast growth factor, α-SMA—αsmooth muscle actin, MMP—matrix metalloproteinases.

**Table 6:** Clinical trials and updates of stem cell-derived exosomes for wound healing and skin regeneration.

Stem Cells Source	Indication	Trial ID	Phase/Status	Administration Route	Outcomes
Wharton’s Jelly Mesenchymal Stem Cells	Chronic Ulcerative Wounds	NCT04134676	Phase I/completed	Topical or Intradermal	Safety, tolerability, and preliminary efficacy in wound healing
Bone Marrow Mesenchymal Stem Cells	Diabetic Cutaneous Ulcers	NCT05243368	Phase I/ongoing	Combined systemic therapy approach	MSC exosome efficacy combined with nutritional correction in diabetic patients
Adipose Tissue Mesenchymal Stem Cells	Chronic Wounds and Wound Repair	NCT05475418	Pilot/ongoing	Topical application via hydrogel dressing	Safety and preliminary efficacy of exosome-loaded hydrogel composite
Platelet-Derived Stem Cell Precursors	Diabetic Foot Ulcers	NCT06319287	Phase 2a/ongoing	Topical application with standard of care	Safety and efficacy with up to 12 weekly applications; wound closure rates compared to standard care
Umbilical Cord Wharton’s Jelly Mesenchymal Stem Cells	Chronic Diabetic Foot Ulcers	NCT06812637	Phase II/ongoing	Topical gel application once weekly for 4 weeks	Percent wound closure and ulcer healing rate at 20 weeks; assessment of scar reduction and tissue quality

## Data Availability

No new data were created or analyzed in this study.
